# Scalpel vs Electrocautery for Upper Lateral Cartilage Contouring in Dorsal Preservation Rhinoplasty: A Retrospective Comparative Study

**DOI:** 10.1093/asj/sjaf180

**Published:** 2025-09-16

**Authors:** Serhat Şibar, Ayhan Işık Erdal, Mustafa Talha Okutan

## Abstract

**Background:**

Dorsal preservation rhinoplasty maintains dorsum integrity while refining nasal aesthetics, but dorsal hump recurrence is a common limitation, especially after extensive reshaping. Electrocautery offers precise cartilage contouring, yet its role in dorsal preservation rhinoplasty is underinvestigated.

**Objectives:**

To compare the outcomes of scalpel-based mechanical reshaping vs electrocautery-assisted thermal reshaping of the upper lateral cartilage shoulders in low septal strip dorsal preservation rhinoplasty.

**Methods:**

The authors of this retrospective study included 205 patients who underwent low septal strip dorsal preservation rhinoplasty via the open approach between February 2021 and May 2023. Patients were grouped according to the method used for reshaping the upper lateral cartilage: Group I underwent mechanical reshaping with a scalpel (mechanical/scalpel group), and Group II underwent thermal reshaping using monopolar electrocautery (thermal/electrocautery group). Dorsal hump recurrence and patient-reported outcomes were evaluated using standardized 12-month postoperative photographs and the Rhinoplasty Outcome Evaluation (ROE) questionnaire, respectively.

**Results:**

A total of 88 patients were included in the scalpel group and 117 in the electrocautery group. Demographic data, hump morphology, and amount of hump reduction were similar between groups. However, the recurrence rate of the dorsal hump was significantly lower in the electrocautery group (2.5%) than in the scalpel group (13.6%). ROE scores were high in both groups (84.4 vs 85.0, *P* > .05).

**Conclusions:**

Electrocautery-assisted upper lateral cartilage reshaping in dorsal preservation rhinoplasty offers more consistent contouring and reduced recurrence rates compared with the scalpel-based technique. It represents a valuable technical adjunct, especially in patients with a challenging dorsal anatomy.

**Level of Evidence: 3 (Therapeutic):**

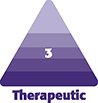

Rhinoplasty is currently among the most commonly performed procedures in facial aesthetic surgery.^1,2^ Problems involving the nasal dorsum—particularly dorsal hump deformities—are among the most frequently cited reasons for surgical correction.^3^ Dorsal hump reduction can be achieved using 2 main approaches. The first is the classical dorsal reduction technique, which involves a resection of both bony and cartilaginous components of the nasal dorsum. Originally described by Joseph, this method requires the disruption and reconstruction of the keystone area and midvault architecture.^4^ Its main advantages include technical simplicity, ease of execution, and effective dorsal reduction. However, potential disadvantages include dorsal irregularities, asymmetry, and complications related to internal nasal valve collapse.

Techniques that aim to preserve the dorsal lines while correcting the hump were first described more than a century ago by Goodale and Lothrop.^5,6^ Over time, approaches that preserve the dorsum structure have been used less frequently because of their technical difficulty and the refinement of the technique described by Joseph with additional surgical maneuvers. In 2018, Saban et al re-evaluated and modernized the technique, leading to its renewed popularity.^7^ Since then, various modifications and techniques under the umbrella of dorsal preservation have been described. Despite their diversity, the most common and characteristic complication among these is hump recurrence.^8^ Tuncel and Aydogdu proposed a terminological distinction between “residual” and “recurrent” hump deformities.^9^ This classification is clinically relevant, because it helps identify etiological factors and implement preventive strategies. Residual humps are generally associated with intraoperative under-resection or masking due to edema and typically become apparent within the early postoperative period (1-3 months). With increasing surgical experience and improved intraoperative assessment, most of these cases can be avoided. On the other hand, recurrent hump typically becomes apparent later in the postoperative period (>3 months) and is attributed to the nasal dorsum's gradual return toward its original position. This is primarily caused by tensile forces acting during healing, which result in the so-called spring effect, transforming the dorsum from a surgically induced concave shape back to a convex contour. Goksel et al described key anatomical factors that may contribute to recurrence in dorsal preservation rhinoplasty, along with various preventive strategies.^10^ Despite thorough management of all relevant anatomical structures in low septal strip dorsal preservation rhinoplasty, the shoulders of the upper lateral cartilages may become convex toward the end of the procedure because of an anterocaudal rotation of the septum. This prominence tends to become more noticeable following major hump reductions, likely as a result of the midvault widening phenomenon known as the “flare effect.” These convexities can usually be corrected intraoperatively using a scalpel or electrocautery without opening the midvault mucosa. However, due to their deep anatomical location, precise contouring of this region can often be technically challenging. Electrocautery provides a practical solution for such cases, offering controlled, rapid, and accurate cartilage reshaping. Although this technique is widely adopted by rhinoplasty surgeons, there remains a scarcity of authors of well-designed studies specifically investigating its effectiveness in dorsal preservation. According to the 2023 Aesthetic Plastic Surgery National Databank Statistics, rhinoplasty consistently ranks among the top 10 aesthetic surgical procedures, holding the ninth place in recent years, with a modest 3.4% increase in procedure volume compared with 2019. This stable yet substantial annual demand underscores the importance of refining surgical techniques to enhance predictability, long-term stability, and patient satisfaction.^11^ The recent concept of advanced preservation rhinoplasty integrates osteoplasty and chondroplasty techniques, expanding the indications for preservation approaches and offering new tools for precise dorsal contour management.^12^

The authors of this study aim to evaluate the efficacy of electrocautery-assisted thermal reshaping of the upper lateral cartilage in patients undergoing low septal strip dorsal preservation rhinoplasty. Technical details and the aesthetic outcomes of electrocautery-assisted thermal reshaping are compared with those of conventional mechanical sculpting using a scalpel.

## METHODS

Following approval by the Clinical Research Ethics Committee of Gazi University (G.U.ET 2024/1439), a retrospective review was conducted of medical records and digital images of patients who underwent open technique low septal strip dorsal preservation rhinoplasty by a single surgeon between February 2021 and May 2023. After applying exclusion criteria—including secondary or revision rhinoplasty, preservation procedures performed without a low septal strip or dorsal impaction, patients diagnosed with a “residual hump” during follow-up, follow-up periods of <1 year, or missing of operative records or photographs—a total of 205 patients were included in the study.

Initially, the senior author performed dorsal reshaping using a scalpel (February 2021–April 2022). From April 2022 to May 2023, the same procedure was carried out using monopolar electrocautery. Based on the technique used for reshaping the upper lateral cartilage, patients were divided into 2 groups: (I) mechanical reshaping with a scalpel (mechanical/scalpel group), (II) thermal reshaping using monopolar electrocautery (thermal/electrocautery group). Thus, the allocation of patients into groups was strictly determined by the date of surgery, ensuring that no case-by-case selection occurred that might introduce selection bias. All patients were evaluated for the presence of dorsal hump using standardized frontal, oblique, and lateral photographs taken preoperatively and at the 12-month postoperative visit. The assessment was performed by a blinded plastic surgeon. All photographs were captured using a Canon EOS 700D camera equipped with a 100 mm macro lens (Canon Inc., Tokyo, Japan).

To evaluate postoperative patient satisfaction, the rhinoplasty outcome evaluation (ROE) questionnaire was administered to all patients during their 12-month postoperative visit. The ROE consists of 6 items: 1 evaluating nasal function and 5 evaluating nasal aesthetics and patient perception. Each item is scored from 0 to 4, and the total score is calculated out of 24 and then normalized to a 100-point scale. Higher scores indicate greater patient satisfaction. A score above 80 was considered to represent a satisfactory outcome.^13^

### Surgical Procedure

All procedures were performed under general anesthesia. Following local infiltration with lidocaine and epinephrine combination, a transcolumellar inverted V incision, combined with marginal incisions, was made to expose the nasal tip, dorsum, and septum. Maneuvers related to the nasal tip—including sliding alar cartilage flap, cephalic trim, lateral crural steal, and medial crural overlay—were completed before dorsal modification to set the desired tip projection and rotation while all structures were stable, because subsequent septal work and low septal strip maneuvers increase mobility and make fine adjustments less predictable. In all cases, the bony cap was excised using either an osteotome or bone scissors, allowing the cartilaginous dorsum to become dominant. This step was incorporated into our standardized low septal strip protocol to optimize dorsal mobility and contour control, particularly in patients with substantial hump reduction, while maintaining the preservation of the keystone area and middle vault continuity. Subperichondrial dissection of the septum was performed, followed by low septal strip septoplasty. Radix and transverse osteotomies were carried out using a microsaw, whereas lateral osteotomies were completed with a conventional osteotome. In cases requiring let-down (eg, hump reduction >4 mm or leaning nose), segmental bone removal from the piriform aperture was performed.

Following impaction, all potential resistance points (eg, Webster's triangle, nasal mucoperichondrium, ethmoid plate) were reassessed. Where necessary, additional dissection or resection was carried out to eliminate resistance. After confirming adequate dorsal reduction, the excess part of the caudal septum was resected. The nasal septum was secured to a notch created on the anterior nasal spine using 2 figure-of-eight 4-0 polypropylene sutures. For tip support, one of the rigid fixation methods—either tongue-in-groove or septal extension graft—was used in all patients. Following septal and tip fixation, the dorsal-tip transition was reassessed.

**Figure 1.**
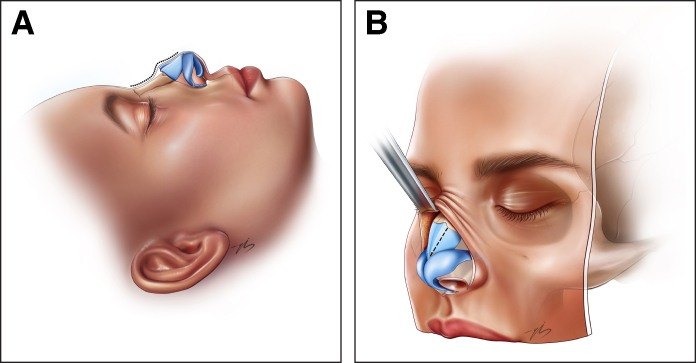
Illustration of the nasal dorsum before thermal contouring. (A) Lateral and (B) oblique views demonstrating the dorsal contour following bony cap excision. The convexity of the upper lateral cartilage shoulders is clearly visible. The dashed contour line delineates the region targeted for subsequent cartilage reshaping. Illustration created by Ezgi Sena Bozoğullarından.

**Figure 2.**
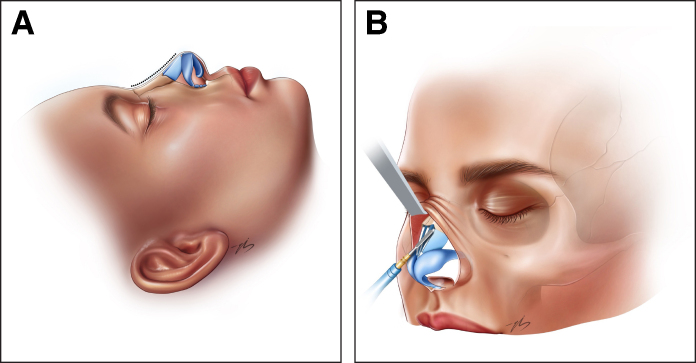
Postoperative appearance of the nasal dorsum following thermal contouring. (A) Lateral and (B) oblique views illustrating the refined nasal dorsum after electrocautery-based reshaping of the upper lateral cartilage shoulder. A smooth contour from the radix to the supratip is observed, with no residual convexity at the midvault level. Illustration created by Ezgi Sena Bozoğullarından.

Before dorsal reshaping was initiated, the mucosal layer underlying the upper lateral cartilages was carefully dissected and fully separated from the cartilage in all patients. This step was routinely performed to prevent mucosal tearing during sculpting and to ensure controlled mobilization of the dorsum. Following the completion of hump reduction, the prominent convex areas at the shoulder of the upper lateral cartilages were reduced gradually. This maneuver was indicated in patients in whom the upper lateral cartilage shoulders were found to be prominent or asymmetric following hump reduction, with the aim of refining the dorsal contour within the dorsal preservation framework. In mechanically reshaped patients, sculpting was performed using a No. 11 scalpel blade. In thermally reshaped patients, needle-tip monopolar electrocautery was used at low-power settings (15 W, cutting/blend) under continuous irrigation with cold (iced) saline. At the end of the procedure, once all dorsal modifications were completed, any minor surface irregularities were corrected by injecting small amounts of autologous diced cartilage beneath the soft tissue envelope to smooth the dorsal contour and ensure a seamless transition. Once the desired dorsal and tip contours were confirmed on lateral and oblique views, the incisions were closed, and both an internal silicone nasal splint (Doyle Splint) and an external splint were applied (Figures 1, 2; Video).

### Power Analysis

A power analysis was conducted using G*Power 3.1 software to determine whether the sample size was adequate to detect statistically significant differences between the 2 groups in terms of demographic variables, dorsal parameters (hump type and degree of reduction), complication rates, and follow-up durations. Analyses were performed separately for continuous variables (eg, age, follow-up time, dorsal reduction) and categorical variables (eg, complication rates).

### Demographics, Dorsal Parameters, and Follow-Up Duration

An independent samples *t* test was used to compare the means of continuous variables between the 2 groups. A medium effect size (Cohen's *d* = 0.5) was assumed, with a type I error probability set at 0.05 and a desired power level of 0.80. The power analysis indicated that a total sample size of 102 patients (51 per group) would be necessary to detect statistically significant differences in continuous variables.

### Complication Rates

The *χ*^2^ goodness-of-fit test was used to compare complication rates between the 2 groups. For categorical variables, a medium effect size (*w* = 0.3) was assumed, with an alpha value of .05 and a desired power of 0.80. The analysis revealed that a minimum of 143 patients was required to achieve adequate power for the *χ*^2^ test. Based on the power calculations, the final sample size of 205 patients was determined to be sufficient for detecting statistically significant differences between the groups for both continuous and categorical outcomes.

### Statistical Analysis

Continuous variables were presented as mean, standard deviation, median, minimum, and maximum values. Categorical variables were summarized using descriptive statistics, including frequency and percentage. The normality of distribution for continuous variables was assessed using the Shapiro–Wilk test and visualized using box plots.

For continuous variables showing normal distribution (eg, age, follow-up duration, ROE score), comparisons between the 2 groups were performed using the independent samples *t* test (Welch's *t* test). For categorical variables, comparisons were made using the *χ*^2^ test or Fisher's exact test when appropriate. A *P*-value <.05 was considered statistically significant.

## RESULTS

All patients were discharged on the day after surgery. Nasal splints were removed on postoperative Day 7. Among the 205 patients included in the study, 151 were female and 54 were male. The shoulders of the upper lateral cartilages were shaped mechanically in 88 patients and thermally in 117 patients. The mean age was 27.8 years (range, 17-49) in the mechanical group and 27.3 years (range, 17-58) in the thermal group. There were no statistically significant differences between the 2 groups in terms of age or gender distribution (*P* > .05).

In the mechanical group, the dorsal hump morphology was V-shaped in 66% and S-shaped in 34% of patients. In the thermal group, 59% had a V-shaped hump and 41% had an S-shaped hump. The amount of dorsal hump reduction was <4 mm in 43.2% and >4 mm in 56.8% of the mechanical group, compared with 46.2% and 53.8%, respectively, in the thermal group. No statistically significant differences were observed between the groups regarding hump morphology or reduction amount (*P* > .05).

**Figure 3.**
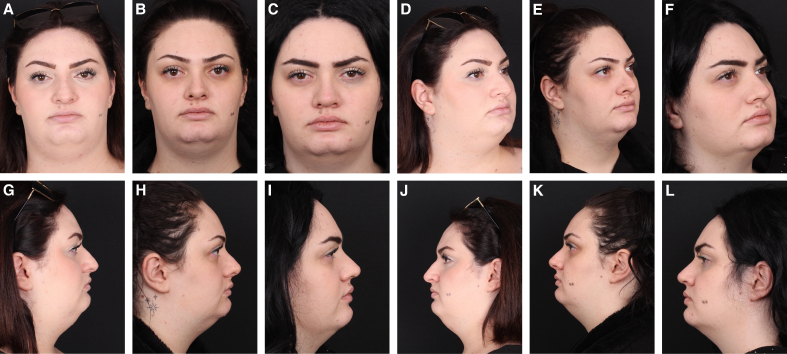
Preoperative (A) frontal, (D) oblique, and (G, J) lateral views of a 26-year-old female patient. (B) Frontal, (E) oblique, and (H, K) lateral views. 14-month postoperative views following mechanical reshaping with a scalpel show a slight convexity at the keystone area. (C) Frontal, (F) oblique, and (I, L) lateral views 8 months after revision surgery show an improved dorsal contour.

**Figure 4.**
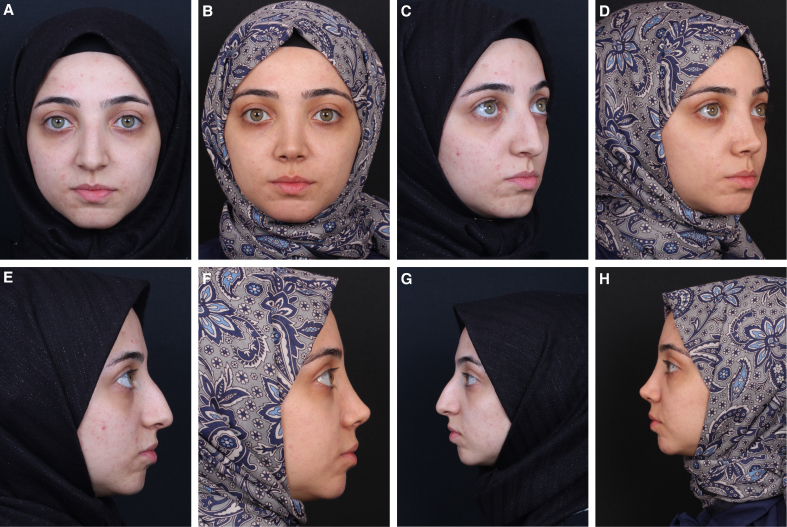
Preoperative (A) frontal, (C) oblique, and (E, G) lateral views of a 22-year-old female patient. Postoperative 15-month (B) frontal, (D) oblique, and (F, H) lateral views following thermal reshaping with electrocautery showing smooth and stable dorsal contours.

**Figure 5.**
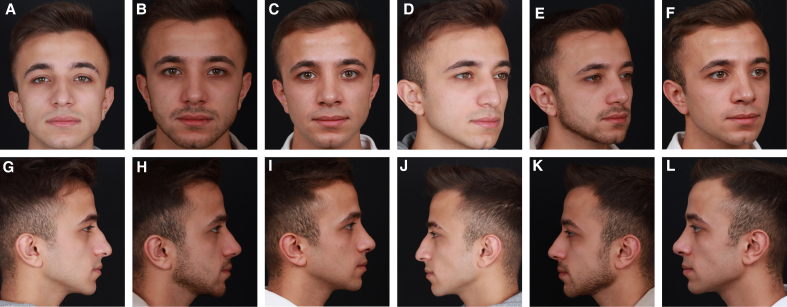
Preoperative (A) frontal, (D) oblique, and (G, J) lateral views of a 23-year-old male patient. Postoperative 12-month (B) frontal, (E) oblique, and (H, K) lateral views following mechanical reshaping with a scalpel demonstrate a slight convexity at the keystone area. (C) Frontal, (F) oblique, and (I, L) lateral views 10 months after revision show an improved dorsal contour.

**Figure 6.**
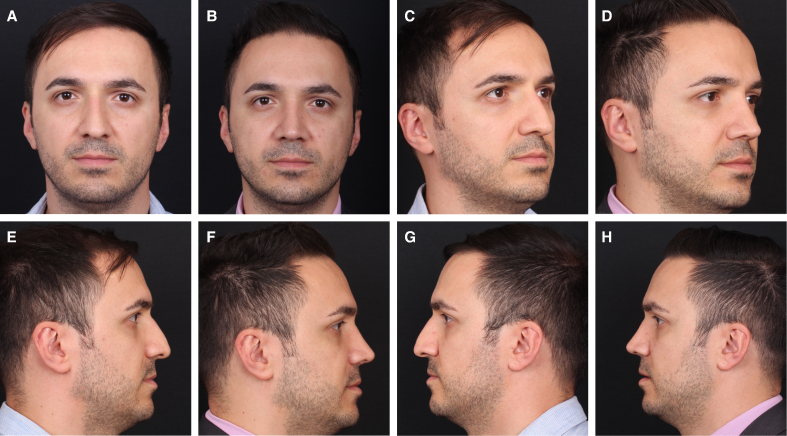
Preoperative (A) frontal, (C) oblique, and (E, G) lateral views of a 35-year-old male patient. Postoperative 12-month (B) frontal, (D) oblique, and (F, H) lateral views following thermal reshaping with electrocautery demonstrate a smooth and well-defined dorsal profile, without evidence of recurrence.

Recurrent dorsal hump was identified in 12 patients (13.6%) in the mechanical group and in 3 patients (2.5%) in the thermal group (Figures 3-6). All patients with recurrence underwent revision surgery. In these cases, the shoulder region of the upper lateral cartilages was reshaped intraoperatively. The mean follow-up period was 20.8 months (range, 12-34 months) in the mechanical group and 14.7 months (range, 12-24 months) in the thermal group. There was a statistically significant difference between the groups in terms of complication rates and follow-up durations (*P* < .05).

The mean ROE score was 84.4 (range, 77.4-91.1) in the mechanical group and 85.0 (range, 79.3-91.8) in the thermal group. The difference between the groups was not statistically significant (*P* > .05) (Table 1).

**Table 1.** Comparative Analysis of Demographic Characteristics, Dorsal Parameters, and Complications Between the 2 Groups

Groups	*n*	Mean age (range)	Gender (F/M)	Hump morphology (V/S)	Amount of hump reduction (<4 mm/≥4 mm)	Dorsal complications (*n*, %)	Mean follow-up (range)
Group I (mechanical reshaping)	88	27.8 (17-49)	67/21	58/30	38/50	12 (13.6%)	20.8 months (12-34)
Group II (thermal reshaping)	117	27.3 (17-58)	84/33	69/48	54/63	3 (2.5%)	14.7 months (12-24)
*P*-value	—	>.05^a^	>.05^b^	>.05^b^	>.05^b^	<.05^b^	<.05^a^

^a^*T* test. ^b^Chi-square test.

## DISCUSSION

The resurgence of dorsal preservation rhinoplasty in recent years has also brought renewed attention to its technical limitations and the search for effective solutions. One of the most characteristic complications associated with this technique is the development of residual or recurrent dorsal hump deformities.^14^ Several maneuvers have been proposed in the literature to prevent these deformities, which may appear in the acute (residual hump) or subacute (recurrent hump) postoperative period.^10,15,16^ In the present study, patients diagnosed with residual hump—defined as deformities observed within the first 1 to 3 months postoperatively—were excluded. This exclusion criterion was designed to minimize confounding factors such as intraoperative assessment error or postoperative edema, which are independent of the reshaping technique itself. By focusing exclusively on recurrent humps that emerge after the third postoperative month, the authors of this study aimed to provide a more accurate comparison between mechanical and thermal reshaping methods in terms of long-term dorsal stability. Careful assessment and management of any anatomical factors that may prevent the dorsum from maintaining its corrected position—or may promote a return to its preoperative shape—are critical for surgical success. Even when the septum is properly positioned, minor or major modifications to the dorsal surface are frequently required. In particular, the bony cap excision and anterior–inferior septal rotation may lead to the flare effect and increased convexity in the shoulders of the upper lateral cartilages. This convexity often distorts the oblique and lateral nasal profiles, necessitating targeted contouring of the dorsum. Dorsal reshaping can be achieved using various techniques, among which the scalpel and electrocautery are 2 commonly employed options. Mechanical reshaping using a scalpel is a well-established method; however, due to the pressure applied directly to the cartilage, it has certain limitations. These include the risk of over- or under-resection, damage to surrounding anatomic structures, and reduced precision in contouring.^17,18^ In dorsal preservation rhinoplasty, the semimobile nature of the dorsum makes it even more challenging to achieve controlled reshaping using a scalpel.

Cartilage reshaping with electrocautery is achieved by transferring thermal energy directly to the cartilage tissue via the heating element embedded within the cautery probe.^19^ Thermal energy affects cartilage by altering the proteoglycan matrix and reorganizing collagen fibers. Several orthopedic studies have demonstrated that the application of thermal energy to articular cartilage results in surface stabilization, producing a macroscopically smoother cartilage surface.^20,21^ Among the main advantages of electrocautery are that it does not require mechanical pressure on the cartilage and allows for more precise, rapid, and controlled contouring, especially in anatomically restricted areas.^17,22^ One of the few studies in the literature evaluating the use of electrocautery for cartilage reshaping in rhinoplasty was conducted by Sella.^22^ In their report, electrocautery was described as an effective, safe, and accessible method, particularly for correcting minor dorsal irregularities. The authors emphasized that electrocautery offers greater precision than conventional scalpel-based contouring. However, their study was limited to a qualitative description of the technique and did not include comparative data such as patient numbers, follow-up duration, or complication rates. In contrast, the present study is among the first retrospective series in which its authors provide quantitative evidence on this topic. Our findings suggest that thermal reshaping is associated with a lower rate of recurrent dorsal hump and more consistent aesthetic outcomes compared with the mechanical method.

These findings are in agreement with recent descriptions by Ferreira et al within the framework of advanced preservation rhinoplasty, in which low-energy electrocautery chondroplasty has been advocated for contour refinement of prominent or asymmetric upper lateral cartilage shoulders, often combined with interpositional absorbable materials to minimize fibrosis during healing. This approach reflects the modern integration of osteoplasty and chondroplasty techniques to enhance dorsal contour stability, while preserving the middle vault.^12^

The primary disadvantage of electrocautery is the risk of chondrocyte necrosis at high temperatures. Although the effect of thermal energy on cartilage viability remains a topic of debate, existing clinical and histological studies have reported that electrocautery can be used safely in rhinoplasty procedures without causing significant chondrocyte loss or tissue necrosis.^17,21-23^ Eravci et al previously used electrocautery to harvest and shape costal cartilage grafts in rhinoplasty, describing the method as “thermal chondroplasty.” They reported that the use of electrocautery reduced graft harvesting time and was associated with fewer complications. In their study, they also suggested that electrocautery may be beneficial for reshaping other cartilage structures within the nose.^17^

The superficial dorsal modification technique, as described by Zholtikov et al, highlights the effectiveness of conservative interventions that reshape the nasal dorsum without altering its dimensions.^24^ In their approach, the shoulders of the upper lateral cartilages were sculpted using either electrocautery or a scalpel, without disrupting the underlying mucosa. In most patients, stable dorsal contours were achieved without the need for spreader grafts. The authors defined this method as a fast, safe, and predictable technique that avoids the need for structural reconstruction. Although our study is grounded in a similar surgical philosophy, the electrocautery-assisted reshaping technique that we used provides a rare quantitative contribution to the literature by comparing recurrence rates and patient-reported satisfaction between thermal and mechanical methods. In our clinical experience, all patients treated with electrocautery developed a thin carbonized (char) layer on the surface of the reshaped cartilage. Authors of previous studies have suggested that this layer may serve as a mechanical barrier, enhancing cartilage surface resistance and protecting the underlying chondrocytes from the detrimental effects of thermal energy.^25,26^ In our series, no complications attributable to thermal injury were observed in the electrocautery group during the follow-up period. This may be attributed to the use of a low-energy mode, delivered intermittently and combined with continuous irrigation using cold saline. Another disadvantage of thermal reshaping, compared with mechanical contouring, is the inability to preserve the integrity of the trimmed cartilage, rendering it unsuitable for grafting purposes. This may be particularly concerning in patients with limited septal cartilage reserves or in patients in whom multiple structural grafts are anticipated. In such scenarios, mechanical contouring techniques may offer the additional advantage of harvesting viable graft material during dorsal reshaping, and thermal techniques should be used with this limitation in mind.

In the literature, the incidence of residual or recurrent dorsal hump following dorsal preservation rhinoplasty has been reported to range between 1% and 15%.^7,9,16,27-29^ In this study, dorsal hump rates in both groups of patients were found to be within the reported range. In patients in whom predominantly bony or kyphotic humps are noticed, several surgical maneuvers can help reduce the likelihood of recurrence. These include resection of the bony cap and nasal bone resections, elimination of all anatomical structures that may resist dorsal impaction, dual-point fixation of the septum, including both anterior nasal spine and posterior base anchoring, and overall rigid stabilization of the septal framework.^30^ When the desired dorsal profile cannot be achieved through these maneuvers alone, direct modification of the dorsal surface should be considered. The significantly lower rate of dorsal hump recurrence in the electrocautery group suggests that thermal reshaping may offer more effective and consistent outcomes in achieving the final dorsal contour compared with mechanical methods. In our protocol, complete removal of the bony cap was performed within a dorsal preservation framework to eliminate potential bony resistance points, particularly in patients with major hump reduction. This was complemented by low-power electrocautery chondroplasty of the upper lateral cartilage shoulders to refine cartilaginous convexities. The combination of these steps aimed to reduce postoperative hump recurrence, while preserving the keystone area.

Electrocautery appears to be particularly effective for addressing convexities in the upper lateral cartilage shoulders. However, in patients in whom dorsal irregularities involve bony components—such as a hypertrophic dorsal hump—powered instruments (eg, surgical burrs or piezoelectric devices) may still be necessary for achieving a satisfactory reduction.

In this study, ROE scores indicated high levels of patient satisfaction in both groups. This may be attributed to the self-reported and subjective nature of the ROE questionnaire, which may not be sensitive enough to reflect subtle differences in surgical techniques as perceived by the patient. Furthermore, although the complication rates differed significantly between the 2 groups, ROE scores are likely more responsive to aesthetic outcomes and overall satisfaction. As such, this difference in complication rates may not have had a noticeable impact on patient-reported satisfaction scores.

### Limitations

Although there were no statistically significant differences between the 2 patient groups in terms of hump morphology or amount of reduction, the retrospective nature of this study limits the ability to control for all potential confounding variables that may affect hump recurrence. In addition, there was a statistically significant difference in mean follow-up duration between the groups, which could potentially influence the recurrence rates observed. Although electrocautery appeared to yield more favorable outcomes, it would be misleading to attribute this effect solely to the reshaping method without considering other technical or anatomical factors.

In addition, although all patients underwent standard bony cap excision as part of the dorsal preservation protocol, the comparative analysis was limited to dorsal convexities that were clinically judged intraoperatively to be cartilage-dominant, based on surgical inspection and revision findings. In all revision cases, the reshaping required was confined to the upper lateral cartilage shoulders, supporting the interpretation that the primary contour irregularities analyzed were not osseous in nature.

Nevertheless, it remains inherently difficult to fully standardize all contributing variables in comparative surgical studies. Future prospective, randomized controlled trials incorporating multivariate analysis are needed to confirm these findings.

## CONCLUSIONS

The authors of this study compared the effectiveness of thermal vs mechanical methods for nasal dorsum contouring in low septal strip dorsal preservation rhinoplasty. Through the results, the authors demonstrated that thermal reshaping using electrocautery was associated with lower complication rates and more consistent aesthetic outcomes. In particular, the rate of recurrent hump deformity was significantly lower in the thermal group. The ability of electrocautery to provide precise and rapid contouring appears especially advantageous in reshaping the semimobile nasal dorsum. When used carefully—at low energy settings and under continuous irrigation—this method offers a safe and effective option for contour refinement. The findings of this study support the use of electrocautery as a reliable alternative technique in dorsal preservation rhinoplasty.

## References

[1] ISAPS. International Survey on Aesthetic/Cosmetic Procedures performed in 2023. Accessed August 3, 2024. https://www.isaps.org/discover/about-isaps/global-statistics/reports-and-press-releases/global-survey-2023-full-report-and-press-releases/

[2] ASPS. 2023 ASPS procedural statistics release. Accessed August 3, 2024. https://www.plasticsurgery.org/news/plastic-surgery-statistics

[3] Chauhan N, Alexander AJ, Sepehr A, Adamson PA. Patient complaints with primary versus revision rhinoplasty: analysis and practice implications. Aesthet Surg J. 2011;31:775–780. doi: 10.1177/1090820X11417427 21908809

[4] Joseph J. The classic reprint: nasal reductions. Plast Reconstr Surg. 1971;47:79–81. doi: 10.1097/00006534-197101000-00015 4921911

[5] Goodale J. A new method for the operative correction of exaggerated Roman nose. Boston Med Surg J. 1899;140:112–112. doi: 10.1056/NEJM189902021400503

[6] Lothrop OA. An operation for correcting the aquiline nasal deformity; the use of a new instrument; report of a case. Boston Med Surg J. 1914;170:835–837. doi: 10.1056/NEJM191405281702205

[7] Saban Y, Daniel RK, Polselli R, Trapasso M, Palhazi P. Dorsal preservation: the push down technique reassessed. Aesthet Surg J. 2018;38:117–131. doi: 10.1093/asj/sjx180 29319787

[8] Cason RW, Goksel A, Sergesketter AR, Cotofana S, Rohrich RJ. Preventing hump recurrence in dorsal preservation rhinoplasty: the five key tenets. Plast Reconstr Surg. 2025;155:2025, 508e–516e. doi: 10.1097/prs.0000000000011528 38722588

[9] Tuncel U, Aydogdu O. The probable reasons for dorsal hump problems following let-down/push-down rhinoplasty and solution proposals. Plast Reconstr Surg. 2019;144:378e–385e. doi: 10.1097/prs.0000000000005909 31461007

[10] Goksel A, Cason RW, Tran KN, Rohrich RJ. The blocking points: the keys to consistent success in preservation rhinoplasty. Plast Reconstr Surg. 2024;153:922e–931e. doi: 10.1097/prs.0000000000010851 37337323

[11] Aesthetic plastic surgery national databank statistics 2023. Aesthet Surg J. 2024;44(Suppl 2):1–25. doi: 10.1093/asj/sjae188 39283306

[12] Ferreira MG, Toriumi DM, Stubenitsky B, Kosins AM. Advanced preservation rhinoplasty in the era of osteoplasty and chondroplasty: how have we moved beyond the Cottle technique? Aesthet Surg J. 2023;43:1441–1453. doi: 10.1093/asj/sjad194 37338117

[13] Alsarraf R, Larrabee WF, Anderson S, Murakami CS, Johnson CM. Measuring cosmetic facial plastic surgery outcomes: a pilot study. Arch Facial Plast Surg. 2001;3:198–201. doi: 10.1001/archfaci.3.3.198 11497506

[14] Tham T, Bhuiya S, Wong A, Zhu D, Romo T, Georgolios A. Clinical outcomes in dorsal preservation rhinoplasty: a meta-analysis. Facial Plast Surg Aesthet Med. 2022;24:187–194. doi: 10.1089/fpsam.2021.0312 35172105

[15] Tuncel U, Kurt A, Saban Y. Dorsal preservation surgery: a novel modification for dorsal shaping and hump reduction. Aesthet Surg J. 2022;42:1252–1261. doi: 10.1093/asj/sjac069 35323904

[16] Tuncel U, Aydogdu IO, Kurt A. Reducing dorsal hump recurrence following push down-let down rhinoplasty. Aesthet Surg J. 2021;41:428–437. doi: 10.1093/asj/sjaa145 32492137

[17] Eravci FC, Ceylan A, Yilmaz M. Thermal chondroplasty technique for costal cartilage harvesting and contouring. J Craniofac Surg. 2020;31:843–846. doi: 10.1097/scs.0000000000006093 31895865

[18] Çerçi Özkan A, Bilgili AM. Clean-cut smoothing of the visible cartilage grafts by sanding with a scalpel. J Craniofac Surg. 2019;30:1875–1876. doi: 10.1097/scs.0000000000005508 30985503

[19] Edwards RB, Lu Y, Markel MD. The basic science of thermally assisted chondroplasty. Clin Sports Med. 2002;21:619–647. doi: 10.1016/s0278-5919(02)00020-0 12489294

[20] Kosy JD, Schranz PJ, Toms AD, Eyres KS, Mandalia VI. The use of radiofrequency energy for arthroscopic chondroplasty in the knee. Arthroscopy. 2011;27:695–703. doi: 10.1016/j.arthro.2010.11.058 21663725

[21] Voss JR, Lu Y, Edwards RB, Bogdanske JJ, Markel MD. Effects of thermal energy on chondrocyte viability. Am J Vet Res. 2006;67:1708–1712. doi: 10.2460/ajvr.67.10.1708 17014320

[22] Sella GCP. Use of electrosurgery to refine cartilaginous structures in rhinoplasty. Biomed J Sci Tech Res. 2023;48:39447–39449. doi: 10.29011/2577-1701.1000165

[23] Mitchell ME, Kidd D, Lotto ML, et al Determination of factors influencing tissue effect of thermal chondroplasty: an ex vivo investigation. Arthroscopy. 2006;22:351–355. doi: 10.1016/j.arthro.2006.01.006 16581445

[24] Zholtikov V, Ouerghi R, Kosins A. Dorsal modification: practical applications in rhinoplasty. Aesthet Surg J. 2024;44:1258–1270. doi: 10.1093/asj/sjae148 38992914

[25] Edwards RB, Lu Y, Nho S, Cole BJ, Markel MD. Thermal chondroplasty of chondromalacic human cartilage. An ex vivo comparison of bipolar and monopolar radiofrequency devices. Am J Sports Med. 2002;30:90–97. doi: 10.1177/03635465020300012801 11799002

[26] Cook JL, Marberry KM, Kuroki K, Kenter K. Assessment of cellular, biochemical, and histologic effects of bipolar radiofrequency treatment of canine articular cartilage. Am J Vet Res. 2004;65:604–609. doi: 10.2460/ajvr.2004.65.604 15141880

[27] Saman M, Saban Y. Long-term follow-up with dorsal preservation rhinoplasty. Facial Plast Surg Clin North Am. 2023;31:13–24. doi: 10.1016/j.fsc.2022.08.004 36396283

[28] Ishida J, Ishida LC, Ishida LH, Vieira JC, Ferreira MC. Treatment of the nasal hump with preservation of the cartilaginous framework. Plast Reconstr Surg. 1999;103:1729–1733; discussion 1734-1735. doi: 10.1097/00006534-199905000-00028 10323714

[29] Wells MW, DeLeonibus A, Barzallo D, Chang IA, Swanson M, Guyuron B. Exploring the resurgence of the preservation rhinoplasty: a systematic literature review. Aesthetic Plast Surg. 2023;47:1488–1493. doi: 10.1007/s00266-023-03345-8 37130993

[30] Çelik V, Tuluy Y. Modification of low-septal-strip septoplasty to reduce hump recurrence in dorsal preservation rhinoplasty. Plast Reconstr Surg. 2025;155:448–456. doi: 10.1097/prs.0000000000011640 39023572

